# A Cross-Sectional Study on Mediating Effect of Chronic Pain on the Relationship between Cervical Proprioception and Functional Balance in Elderly Individuals with Chronic Neck Pain: Mediation Analysis Study

**DOI:** 10.3390/jcm12093140

**Published:** 2023-04-26

**Authors:** Abdullah Raizah, Ravi Shankar Reddy, Mastour Saeed Alshahrani, Ajay Prashad Gautam, Batool Abdulelah Alkhamis, Venkata Nagaraj Kakaraparthi, Irshad Ahmad, Praveen Kumar Kandakurti, Mohammad A. ALMohiza

**Affiliations:** 1 Department of Orthopaedics, College of Medicine, King Khalid University, Abha 61421, Saudi Arabia; 2 Department of Medical Rehabilitation Sciences, College of Applied Medical Sciences, King Khalid University, Abha 61421, Saudi Arabia; 3 College of Health Sciences, Gulf Medical University, Ajman 4184, United Arab Emirates; 4 Department of Rehabilitation Sciences, College of Applied Medical Sciences, King Saud University, Riyadh 11362, Saudi Arabia

**Keywords:** position sense, neck pain, chronic pain, proprioception, balance

## Abstract

(1) Background: Cervical proprioception maintains head orientation in space and contributes to maintaining bodily balance. Evaluating cervical proprioception in elderly individuals with chronic neck pain (CNP) and understanding how pain intensity mediates the relationship between proprioception and functional balance helps formulate treatment strategies for this population. The objectives of this study are to (a) compare the cervical proprioception and functional balance between CNP and asymptomatic, (b) investigate the relationship between cervical proprioception and functional balance ability in CNP individuals and (c) mediation effect of chronic pain on the relationship between cervical proprioception and functional balance tests (2) Methods: This cross-sectional comparative study recruited 60 elderly individuals with a diagnosis of CNP (mean age: 66.40 years) and 60 asymptomatic (mean age: 66.42 years). The cervical proprioception is measured using the target head repositing technique. The subjects were asked to close their eyes and reposition their head actively to the target position from the neutral position, and the reposition accuracy is estimated as joint position errors (JPE) in degrees. The cervical proprioception was measured in the directions of flexion, extension, and left and right rotation. The functional balance was assessed using the berg balance test (BBS) score and timed-up-and-go (TUG) test in seconds. (3) Results: The elderly individuals with CNP had increased cervical JPE compared to the asymptomatic group (*p* < 0.001) in all the directions tested, indicating that cervical proprioception is impaired in CNP patients. Moreover, the CNP individual functional balance is significantly impaired (*p* < 0.001) compared to asymptomatic. The BBS test scores were lower, and the TUG scores were higher in the CNP group. In CNP individuals, the cervical JPE showed a significant correlation with the BBS test scores (r = −0.672 to −0.732, *p* < 0.001) and TUG scores (r = 0.328 to −0.414, *p* < 0.001). (4) Conclusions: Cervical proprioception and functional balance are impaired in elderly individuals with CNP. Physical therapists and rehabilitation professionals may consider these factors during the evaluation and development of treatment strategies in elderly adults with CNP.

## 1. Introduction

In the general population, neck pain is relatively prevalent, and the prevalence of neck pain varies worldwide from 14.6% to 55.2% [1]. It has been estimated that between 12% and 18% of people experience chronic neck pain (CNP), which usually lasts for more than three months [2]. The prevalence of CNP is gradually rising due to factors including modern lifestyle, increased computer usage, and psychological stress [3]. Neck pain and disability substantially influence the healthcare system and impose a financial burden owing to lost workdays [3].

The etiology of CNP is multifactorial, and the most underlying factors are idiopathic [4]. Previous studies have shown a strong relationship between impaired proprioception and the presence and maintenance of chronic pain in different musculoskeletal conditions [2]. Cervical proprioception is vital in maintaining head stability and orientation in relation to the trunk and is a prime fundamental component of proprioception [5]. Different authors have shown a relationship between impaired proprioception and cervical spine injury and recurrence of pain in different cervical pathologies [6,7,8].

Cervical proprioception, in conjunction with the visual and vestibular systems, maintains bodily balance and posture while providing afferent proprioceptive inputs to optimize motor control and fine-tune muscle activation patterns [9]. The cervical spine has abundant muscle spindle density and contributes to a refined and efficient proprioceptive system that controls the neck reflex systems [10]. These reflexes are crucial in maintaining balance, head and eye coordination, and equilibrium (static and dynamic) [11]. In literature, different authors have demonstrated impaired cervical proprioception with increased reposition errors in subjects with different neck syndromes compared to asymptomatic [8,12,13,14]. These investigations suggest that the ensuing somatosensory dysfunction could result in delays and errors in updating the information required to maintain a balance [14]. Moreover, impaired proprioception significantly impacts postural stability in different disease conditions [14,15,16]. It is proposed that proprioception and balance should not be ignored if patients with CNP attain optimal outcomes [17]. 

The fear–avoidance model explains the possible influence of pain on the sensory-motor system and leads to musculoskeletal system modifications, resulting in disability and reduced quality of life [18]. The transformation of acute pain into chronic pain is caused by biopsychological processes and catastrophic thinking, which also explains the possible links between chronic pain and a vicious circle of disability and anguish [19]. Increased pain severity and catastrophizing may be considered a precursor of pain-related fear in CNP patients [20]. Persistent pain can have a deleterious effect on the motor behavior of the cervical spine, consequently affecting the cervico-cephalic kinaesthetic sensibility, as well as the balancing parameters [21]. This aspect suggests that pain may mediate cervical proprioception and body balance in CNP patients, and it is crucial to comprehend their interaction with pain as a mediator. In order to gain a better understanding of the relationship, we utilized mediation analysis with multiple regression [18].

There are no studies that assessed cervical proprioception and functional balance in elderly individuals with CNP and how pain intensity may mediate the relationship between them. Assessing and understanding their relationship will allow clinicians and rehabilitation therapists to formulate treatment strategies and help to manage these patients conservatively. The objectives of this study were to (1) compare the cervical proprioception and functional balance variables between CNP and asymptomatic and (2) investigate the relationship between cervical proprioception and functional balance ability in elderly CNP individuals. The fundamental hypothesis of this study was that proprioception and functional balance would be impaired in elderly individuals with CNP compared to asymptomatic, and cervical proprioception will have a significant association with functional balance in elderly individuals with CNP.

## 2. Materials and Methods

### 2.1. Ethics and Study Design

This observational cross-sectional study was conducted from May 2020 to April 2022 at King Khalid University, Department of Physiotherapy, in cooperation with orthopedic and general medicine clinics. The University’s ethics committee approved the study protocol (REC# 22-10-2020), and the authors ensured that study procedures adhered to the principles outlined in the Declaration of Helsinki for conducting medical research on human participants.

### 2.2. Participants

An orthopedic doctor referred the study participants diagnosed with non-specific CNP to a physiotherapy clinic. Chronic non-specific neck pain is diagnosed as cervical pain without a known pathological basis as the underlying cause of the complaints, and neck pain is caused by postural or mechanical factors [22,23]. Neck pain is considered to be chronic if it lasts longer than three months [24]. Patients with non-specific CNP were considered for inclusion if they fulfilled the following criteria: (1) those who had neck pain without a pathological cause [25], (2) were aged between 60 to 80 years, (3) complained of pain or discomfort in the region extending from the sub-occipital to the first thoracic vertebra for a minimum of 12 weeks [26], and (4) have a visual analog scale (VAS) score of three or higher for the week before the day of assessment [27]. The subjects were excluded if they had a history of trauma, were pregnant, had a history of neurological disorders or vestibular impairment, had a diagnosis of fibromyalgia syndrome or chronic fatigue syndrome, and were unable to follow examiner commands. All the patient’s medical records were examined to rule out any known pathological causes for neck pain. Additionally, all the patients were evaluated using Spurling’s test for positive radiculopathy signs and symptoms and Dix Hallpike maneuvers for positive, benign paroxysmal positional vertigo, and the subjects with positive tests were excluded. All the individuals were independent in walking and did not use any assistive devices for ambulation. Asymptomatic adults were recruited for the study by distributing pamphlets and delivering lectures on the university campus and in nearby communities. Participants met the inclusion criteria if they were in excellent health, between the ages of 60 and 80, and able to comprehend and follow the examiner’s instructions. An experienced physical therapist assessed all the musculoskeletal examinations and the demographic information with a decade of experience post-doctorate. 

### 2.3. Outcome Measures

#### 2.3.1. Pain Assessment

The neck pain intensity of the patients was evaluated using a VAS score [28]. On this scale, 0 represents “no pain”, while 100 mm represents the “worst pain”. The scale ranges from 0 to 100 mm. When determining neck pain intensity, the VAS is a reliable and valid instrument for this population [28]. 

#### 2.3.2. Cervical Joint Position Sense Evaluation

**Figure 1:**
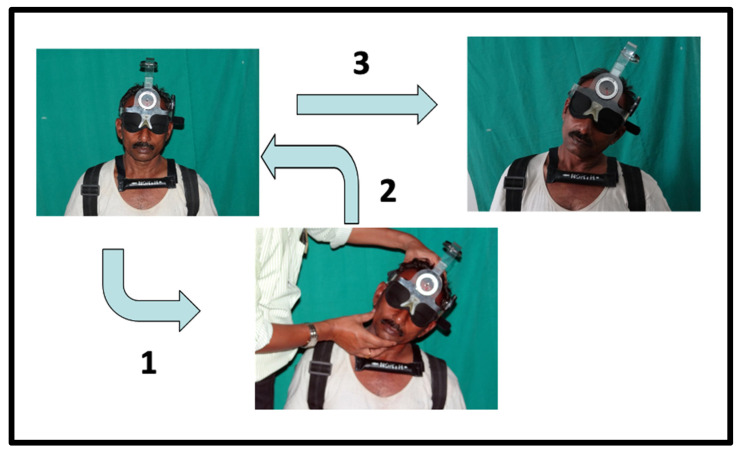
Measurement procedure of cervical proprioception using a cervical range of motion device. Step 1: examiner guides the participant’s neck from the starting position to the target position; step 2: return to the starting position after memorizing the target position; and step 3: repositioning to the target position actively by the participant.

The cervical range of motion (CROM) instrument was utilized to evaluate cervical proprioception in the flexion, extension, and right and left rotation directions [29]. CROM is a valid and reliable instrument for measuring cervical range of motion and proprioception in individuals with and without neck symptoms [29,30,31]. The unit is helmet-shaped, including three inclinometers to measure the range of motion in sagittal and transverse planes (Figure 1). 

The unit comprises a magnetic yoke specifically utilized to limit the influence of thoracic rotation during right and left rotation measurements. The target head position sense was evaluated as a measure of cervical proprioception [32]. This THP technique’s intra-rater reliability varied from good to excellent, with ICC values between 0.70 and 0.83 and SEM between 1.45 and 2.15. Similarly, inter-rater reliability with ICC values between 0.62 and 0.84 and SEM values between 1.50 and 2.23 [32]. The target was 25 degrees of flexion, extension, and right and left rotation. The subject sat on a chair with back straight, knees flexed to 90 degrees, and feet flat on the floor. The subject’s shoulder was fixed with the strap to avoid alternate movements during the proprioception assessment. The subjects were instructed to close their eyes for the proprioception examination. The CROM gadget is attached to the head of the individual using Velcro. The magnetic yoke is positioned around the subject’s shoulder such that its arrow points north. The examiner initiates the target head position examination by passively flexing the subject’s head 25 degrees (Figure 1). After maintaining the position for five seconds, the patient is prompted to remember it. The head is then returned to its neutral position. The patient was then instructed to actively move his neck and reposition himself in the target position. Once the subject is repositioned to the target position, he or she signaled by saying “YES”. The repositioning sense is assessed as JPE in degrees. Similar tests were conducted in the directions of cervical extension and right and left rotation. We measured the Absolute error, which is the overall deviation, without considering the direction. Six JPE test trials were conducted in each movement direction, and the average of these trials was calculated for analysis. The mean JPE score = (sum of 6 trials/6). The greatest test–retest reliability was obtained when cervical JPE was tested six times in each movement direction (ICC = 0.73–0.84) [33].

#### 2.3.3. Berg Balance Scale (BBS) Test

The BBS is designed to objectively assess a patient’s ability to do a series of specified tasks while keeping their balance [34]. It includes a list of daily functional activity tests totaling 14 items, each of which is graded on a five-point ordinal scale ranging from 0 to 4, with 0 denoting the lowest degree of function and 4 denoting the highest level of function, and it a maximum score of 56 points [35]. A score of 41–56 = low fall risk, 21–40 = medium fall risk, and 0–20 = high fall risk. The BBS test is valid, with ICC values between 0.98 and 0.99 [34]. 

#### 2.3.4. Timed Up and Go (TUG) Test

**Figure 2:**
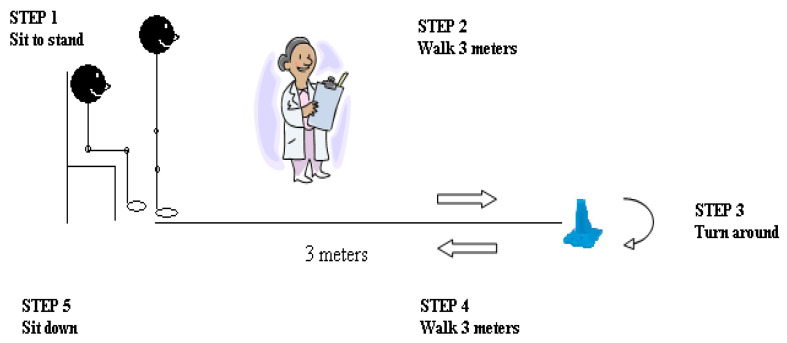
Performance assessment of Timed Up and Go test.

Functional balance is evaluated using the TUG test in elderly individuals. During the TUG test, the participants sat in a typical armchair, got up, walked three meters, turned around, and then got back into the chair and sat down (Figure 2). The total time it took them to complete this task was recorded. The test was repeated thrice, and the average score was used for analysis. With a value of 0.99 for its ICC, the TUG test has a high degree of test–retest reliability [36]. An elderly person who cannot finish the TUG in less than 12 s has an increased chance of falling [37].

An independent blinded investigator with 10 years of experience in the musculoskeletal field evaluated the cervical proprioception and functional balance parameters.

### 2.4. Sample Size Estimation

A sample size calculation (G*Power, version 3.1.6, Heinrich Heine University Düsseldorf, Düsseldorf, Germany) was utilized to provide an appropriate sample size producing 90% power, an effect size of 0.75, and an alpha set at 0.05. Using the independent test method, the effect size was calculated using the mean cervical joint position error scores and accompanying standard deviations from Alahmari et al. [38]; we require a minimum of 55 participants in each group. To account for the possibility of missing data or dropouts, we enrolled 60 people in each group.

### 2.5. Statistical Analysis

**Figure 3:**
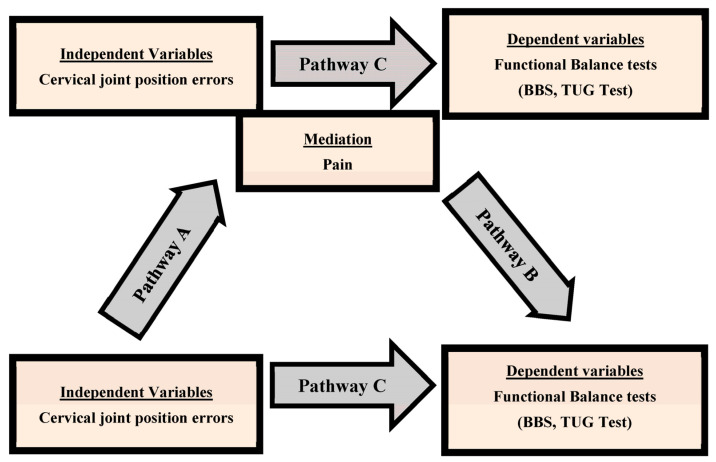
Model of the potential mediating effect of pain on the relationship between cervical proprioception and functional balance parameters.

The Shapiro–Wilk tests were used to assess the data distribution’s normality, and the study variables followed a normal distribution. The study variables are expressed in mean and standard deviation. CNP and asymptomatic groups’ differences in cervical proprioception and balance characteristics were compared using independent *t*-tests. Cohen’s d was utilized to assess the magnitude of the effect (effect size) of between-group differences. The cervical proprioception and balance in CNP patients were correlated using Pearson’s correlation coefficient (r). The correlation was estimated as low (r = 0.20 to 0.30), moderate (r = 0.31 to 0.69), and high (r = 0.70 to 1) [39]. The mediation analysis included a three-step process (Figure 3) [40]. 

Bi-variate regression assessed the total effect between cervical proprioception JPE scores and functional balance tests (step 1). The direct effect between pain and Cervical JPE (pathway A) was assessed using bivariate regression (step 2). Multiple regression was used to assess the direct effect between cervical proprioception and functional balance parameters JPE (Pathway C) and between Cervical JPE and pain (Pathway B) as a step 3. The Sobel test is used to determine whether chronic pain mediates the effect of cervical JPE (independent variable) on the functional balance parameters (dependent variable). A significant test statistic offers evidence that an independent variable has an indirect effect (i.e., an effect that is mediated in whole or in part through another variable) on the dependent variable. A probability level of 5% was deemed statistically significant. Statistical analysis was performed using SPSS version 24.0 (IBM Crop., Chicago, IL, USA). 

## 3. Results

Participants in this study included sixty individuals who had been diagnosed with CNP, as well as sixty asymptomatic individuals. Table 1 provides a summary of the demographic characteristics of the population that was used for the investigation. Compared to the asymptomatic group, the CNP group had a higher body mass index (BMI = 25.67 ± 4.02; *p* < 0.001).

Table 2 summarizes the cervical proprioception and functional balance parameters between the groups.

In all directions examined, the cervical JPE was greater in the CNP group than in the asymptomatic group (*p* < 0.001), showing that cervical proprioception is impaired in CNP patients. In addition, the CNP group has significantly reduced functional balance (*p* < 0.001). The BBS test results were lower in the CNP group, while the TUG test scores were higher (Table 2).

The linear relationship between cervical proprioception and functional balance parameters is summarized in Table 3. 

The cervical proprioception showed a significant negative correlation with the BBS test scores (r = −0.672 to −0.732, *p* < 0.001) in CNP individuals (Table 3). The individuals with larger cervical JPE showed impaired balance. Moreover, cervical proprioception significantly correlated with TUG scores (r = 0.328 to −0.414, *p* < 0.001). Those with greater cervical JPE took longer to complete TUG testing.

The results of the mediation analysis are summarized in Table 4 and Table 5.

As shown in Figure 3, in this mediation model, the total effect was the observed effect of cervical JPE on functional balance (pathway C). Cervical JPE. The total effect also decomposed into the direct effect of cervical JPE on functional balance parameters (pathway C′) and the indirect effects of cervical JPE on functional balance through the pain (mediated: pathway A + B). The indirect effect was statistically significant (Sobel test) that pain explained the association between cervical JPE on functional balance (*p* < 0.05). 

## 4. Discussion

We designed this cross-sectional study to compare cervical proprioception and functional balance parameters between CNP and asymptomatic and to assess the correlation between cervical proprioception and functional balance parameters in CNP individuals. Moreover, we assessed whether pain severity could mediate the relationship between cervical JPE and functional balance parameters. This study showed that cervical proprioception and functional balance are significantly impaired in the CNP group as compared to the asymptomatic group. Cervical proprioception showed significant correlations with functional balance parameters. The pain severity significantly mediated the relationship between cervical JPE and functional balance parameters. 

The results of this study showed that CNP individuals had impaired cervical proprioception compared to asymptomatic. The cervical spine is rich in mechanoreceptors and contributes to afferent proprioceptive input to the higher centers [41,42]. Patients with CNP may have changes in motor activation patterns and reaction timings [43], also a decrease in muscle strength and endurance as a result of the decrease in muscle spindle size and number [44]. Changes in these factors can significantly impact the afferent proprioceptive input to the higher centers and impair the joint position sense [44]. Individuals with CNP exhibit a fear of movement-altering muscle activation strategies and coordination [45]. These features can, along with catastrophic thinking, alter the muscle recruitment patterns, mainly inhibiting the deep segmental muscles and the activation of superficial muscles [46]. The primary source of proprioception afferents in the neck area, as was already established, and muscle spindles that are closely clustered in the deep neck muscles [43]. The discharge of muscle spindles can shift as a result of these anatomical and functional changes in the cervical deep and superficial muscles, which alters afferent input and impacts proprioception [6]. The findings imply that individuals with persistent neck pain experience the complexity and diverse character of neck muscle weakness [47]. Moreover, the impact of pain on various levels of the neural system can vary how sensitive muscle spindles are as well as how the cortex represents and regulates the input from the cervical afferent [48,49]. 

Cervical JPEs varied in magnitude from 5° to 6° in CNP participants in this study, and similar JPE magnitudes were found in CNP participants in earlier published investigations [6,7,50,51,52,53]. Initially, it was by Revel et al. [54] demonstrated that CNP individuals had significantly impaired cervical JPE (6.11° ± 1.34°) compared to asymptomatic (3.50 ± 1.64°) when they were repositioned to the target head position. Studies that evaluated cervical proprioception in different age groups showed that the magnitude of cervical JPE was larger in elderly subjects compared to younger individuals [38,55]. Şekeröz et al. [55] conducted a study to assess the impact of CNP on cervical proprioception in elderly individuals aged 65 years or over and concluded pain significantly impacted cervical proprioceptive sensibility [55]. Alahmari et al. [38] also stated that elderly individuals with CNP over 50 years have larger cervical JPE (6.57° to 7.80°) compared to age-matched asymptomatic (1.95° to 2.95°). Our results might propose that CNP individuals are associated with decreased proprioceptive acuity.

The elderly subjects with CNP had impaired functional balance compared to those asymptomatic. In accordance with our study results, previously published studies showed that balance is impaired in subjects with neck pain compared to asymptomatic [56,57,58]. Jull et al. [59] have attributed this imbalance to abnormal cervical spine proprioceptive input in CNP individuals [59]. An additional theory for impaired functional balance in this population is a mismatch between cervical, visual, and vestibular reflex connections and inappropriate signals conveyed to the central nervous system [14]. There is a positive association between neck pain intensity and functional balance impairments in patients with non-specific neck pain [58]. These results are consistent with those of Uthaikhup et al. [60] and Poole et al. [61], who observed that CNP participants ≥65 years old had impaired balance than the control group. Different authors previously used simple standing position or Romberg standing and could not detect any balance impairments in non-specific CNP individuals [62]. The results of this study demonstrated that the functional balance tests (the BBS test and the TUG test) could detect functional balance impairments in individuals with non-specific CNP that may be concealed under simple static settings.

There was a moderate to strong correlation between cervical JPE and TUG and BBS scores. The individuals with CNP with a higher pain intensity had a higher JPE score and a decreased functional balance score. This correlation suggests that improving cervical proprioceptive ability will improve functional balance and vice versa. Impaired balance is strongly associated with increased falls in elderly individuals [63]. Poor functional balance in this population can be attributed to decrease muscle strength, cognitive dysfunction, and impaired proprioception [64,65]. Future studies should focus on assessing improvements in functional balance parameters following cervical proprioception retraining [66]. 

We anticipated that pain might mediate the relationship between cervical proprioception and functional balance. A review of prior studies could add credence to our hypothesis of pain mediation on motor control. A compilation of study findings revealed a considerable association between pain and chronic musculoskeletal diseases [67,68,69]. Cervical proprioception may be compromised by decreased flexibility, deconditioning, and loss of muscle tone brought on by increased pain, chronicity, and disuse [70]. Pain can influence numerous aspects of the nervous system, including the sensitivity of muscle spindles and the way the central nervous system modulates proprioceptive afferent signals [71]. Asiri et al. [72] conducted a study investigating the mediation effect of pain on the postural control in fibromyalgia syndrome, and the results demonstrated that pain significantly mediated to produce altered motor control, hence impacting balancing ability. Similarly, in our study, the pain may have impacted the relationship between cervical proprioception and balance parameters. Moreover, proprioception may have been significantly impaired by the presence of pain, and the magnitude of impairment has intensified by the chronicity of pain.

### 4.1. Practical Clinical Implications

This study revealed that patients with CNP had decreased proprioception and functional balance compared to asymptomatic individuals, and earlier research has demonstrated that this population falls more frequently [73]. These results corroborate other studies that have suggested individuals with CNP had more frequency of falls and have shown that chronic pain can make patients with CNP more susceptible to balance issues [73]. For individuals with CNP who are undergoing rehabilitation, the study’s findings have clinical ramifications. Moreover, pain severity played a major role in an impaired functional balance associated with CNP. Physical therapists and clinicians should. Consider these factors during the evaluation and formulation of treatment strategies for elderly individuals with CNP.

### 4.2. Limitations

This study has a few limitations. To begin with, although we examined cervical proprioception using a CROM device, a popular tactic of evaluation, it is not the gold standard; computer-based FastTrack devices can provide more precise data. Furthermore, because a cross-sectional study design was adopted, a causal association between the occurrence of a temporal sequence and disease progression could not be demonstrated. However, the first stage of planning a longitudinal study could be the inferences from a cross-sectional study. Even though the functional balance was assessed, information regarding the frequency of falls in CNP individuals was not assessed. As the prospective study design was not adopted, Patients with CNP were not included in a rehabilitation program to address functional balance or cervical proprioception abnormalities, and the outcomes of rehabilitation intervention were not investigated. 

## 5. Conclusions

The subjects with CNP had decreased cervical proprioception and functional balance compared to asymptomatic participants. Cervical proprioception showed a significantly strong relationship with functional balance, indicating individuals with larger cervical JPE had more impaired functional disabilities. Chronic pain experienced by CNP patients significantly mediated the relationship between proprioception and functional balance parameters.

## Figures and Tables

**Table 1:** Physical and demographic characteristics of study participants.

Variables	CNP Individuals (*n* = 60)	Asymptomatic (*n* = 60)	*p*-Value
Age (years)	66.40 ± 4.33	66.42 ± 4.59	0.984
Height (meters)	1.68 ± 0.10	1.73 ± 0.05	0.002
Weight (kg)	71.70 ± 6.33	69.37 ± 5.23	0.030
BMI (kg/m^2^)	25.67 ± 4.02	23.28 ± 2.80	<0.001
Pain intensity: VAS (0–10 cm)	5.92 ± 1.36	-	-

CNP = chronic neck pain, BMI = body mass index, VAS = visual analog scale.

**Table 2:** Comparison of cervical joint position sense and functional balance parameters between chronic neck pain and asymptomatic individuals.

Variables	CNP Individuals (*n* = 60) (Mean ± SD)	Asymptomatic (*n* = 60) (Mean ± SD)	F	*p*-Value
JPE in flexion (°)	5.45 ± 1.42	2.38 ± 1.28	154.82	<0.001
JPE in extension (°)	6.27 ± 1.53	2.98 ± 1.26	165.40	<0.001
JPE in left rotation (°)	5.02 ± 1.31	2.95 ± 0.70	116.45	<0.001
JPE in right rotation (°)	5.40 ± 1.37	2.98 ± 0.23	136.47	<0.001
BBS test score	47.60 ± 4.48	54.12 ± 5.46	851.34	<0.001
TUG test (sec)	10.38 ± 1.46	9.00 ± 1.01	39.24	<0.001

CNP = chronic neck pain, JPE = joint position error, BBS = Berg balance scale, TUG = Timed Up and Go. *p*-values are based on post-hoc Bonferroni correction.

**Table 3:** Relationship between cervical joint position errors and functional balance parameters in CNP individuals (*n* = 60).

Explanatory Variables	BBS Test	TUG Test
JPE in flexion (°)	−0.686 **	0.395 **
JPE in extension (°)	−0.732 **	0.414 **
JPE in left rotation (°)	−0.703 **	0.328 **
JPE in right rotation (°)	−0.672 **	0.364 **

CNP = chronic neck pain, JPE = joint position error, BBS = Berg balance scale, TUG = Timed Up and Go, ** = Correlation is significant at the 0.01 level (2-tailed).

**Table 4:** Mediation analysis using pain as mediation between cervical proprioception and functional balance parameters.

Test Variables	Total Effect—Direct and Indirect			Direct Effect			Indirect Effect		
	B	SE	*p*-Value	B	SE	*p*-Value	B	SE	*p*-Value
Pain × JPE in flexion (°) × BBS	0.32	0.03	0.001	0.13	0.02	<0.001	0.05	0.01	0.002
Pain JPE in extension (°) × BBS	0.43	0.04	0.001	0.20	0.01	<0.001	0.04	0.01	0.002
Pain × JPE in rotation left (°) × BBS	0.28	0.01	0.011	0.16	0.01	<0.001	0.09	0.02	0.003
Pain × JPE in rotation right (°) × BBS	0.26	0.02	0.012	0.14	0.01	<0.001	0.11	0.02	0.004
Pain × JPE in flexion (°) × TUG	0.42	0.04	0.001	0.31	0.02	<0.001	0.05	0.01	0.002
Pain JPE in extension (°) × TUG	0.53	0.03	0.001	0.30	0.01	<0.001	0.04	0.01	0.002
Pain × JPE in rotation left (°) × TUG	0.48	0.02	0.011	0.26	0.01	<0.001	0.09	0.02	0.003
Pain × JPE in rotation right (°) × TUG	0.46	0.03	0.012	0.24	0.01	<0.001	0.11	0.02	0.004

JPE = joint position error, BBS + Berg balance scale, TUG = Timed Up and Go test, B = unstandardized coefficients, SE = standard error.

**Table 5:** Sobel test for indirect effect for statistical significance.

Test Variables	Sobel-Test	SE	*p*-Value
Pain × JPE in flexion (°) × BBS	0.34	0.04	0.040
Pain JPE in extension (°) × BBS	0.44	0.03	0.030
Pain × JPE in rotation left (°) × BBS	0.26	0.02	0.028
Pain × JPE in rotation right (°) × BBS	0.36	0.04	0.029
Pain × JPE in flexion (°) × TUG	0.44	0.10	0.020
Pain JPE in extension (°) × TUG	0.53	0.12	0.010
Pain × JPE in rotation left (°) × TUG	0.35	0.11	0.030
Pain × JPE in rotation right (°) × TUG	0.47	0.09	0.019

JPE = joint position error, BBS = Berg balance scale, TUG = Timed Up and Go test, SE = standard error.

## Data Availability

All data generated or analyzed during this study are with the corresponding author (RSR) and the data will be provided on request.
